# Cytoplasmic changes in bone marrow cells as indicators of postmortem interval.

**DOI:** 10.12688/f1000research.169755.2

**Published:** 2025-12-26

**Authors:** Mahabalesh Shetty, Vinoth Guru, Kishan Prasad HL, Suraj S Shetty

**Affiliations:** 1 Professor, Nitte (Deemed to be University), KS Hegde Medical Academy (KSHEMA), Department of Forensic Medicine and Toxicology, Mangalore, India; 2 Resident, Nitte (Deemed to be University), KS Hegde Medical Academy (KSHEMA), Department of Forensic Medicine and Toxicology, Mangalore, India; 3 Professor, Nitte (Deemed to be University), KS Hegde Medical Academy (KSHEMA), Department of Pathology, Mangalore, India; 4 Professor and Head, Nitte (Deemed to be University), KS Hegde Medical Academy (KSHEMA), Department of Forensic Medicine and Toxicology, Mangalore, India

**Keywords:** Bone Marrow Cytology, Forensic Pathology, Postmortem Interval (PMI)

## Abstract

**Background:**

Accurate estimation of the postmortem interval (PMI) remains one of the central challenges in forensic medicine. Traditional external markers are limited by environmental and individual variability. Bone marrow, owing to its protected anatomical location and slower decomposition rate, offers a potential histological substrate for PMI estimation.

**Aim:**

This study aimed to evaluate cytoplasmic changes in sternal bone marrow cells and assess their correlation with Postmortem interval (PMI).

**Methods:**

A descriptive cross-sectional study of 43 medico-legal autopsies with documented time of death. Sternal bone marrow aspirates were stained with Leishman stain and examined microscopically. Cytoplasmic changes were scored from 0 to 4 (intact morphology to complete dissolution) using a system adapted from Biradar G et al. Data were analyzed using ANOVA (p < 0.05).

**Results:**

Mild cytoplasmic alterations (S1) were most frequent (51.1%), followed by moderate changes (S2, 25.6%) and severe dissolution (S4, 14%). Only 9.3% of cases showed no observable changes (S0). Mean PMI increased with the severity of cytoplasmic changes (S0: 8.5 h; S1: 14.1 h; S2: 10.5 h; S4: 15.0 h), although some overlap was seen, especially at S2, likely reflecting biological and environmental variability. The overall correlation was not statistically significant (p > 0.05).

**Conclusion:**

Cytoplasmic autolysis in bone marrow appears to follow a progressive pattern, from early vacuolation to complete dissolution with increasing PMI. Although not statistically significant, observed trends suggest cytoplasmic scoring may aid early PMI estimation when combined with other markers.

## Introduction

Thanatology, derived from the Greek word *thanatos* (meaning “death”), refers to the scientific study of death and its processes. It represents an interdisciplinary field encompassing significant elements of Anthropology, Pathology, Biochemistry, and Entomology. ^1^

A forensic autopsy is a vital process that helps shed light on the circumstances of deaths that are suspicious, sudden, or legally disputed. It plays a crucial role in uncovering the truth and providing answers for families, investigators, and the judicial system. For forensic pathologists, the primary goals include establishing the identity of the deceased, determining the cause of death, and, importantly, estimating the post-mortem interval (PMI), often referred to as Time Since Death (TSD). ^2^

Traditionally, Time since death (TSD) is calculated by looking at external physical changes such as body cooling (algor mortis), settling of blood (livor mortis), stiffening of muscles (rigor mortis), and the process of decomposition (putrefaction). Although these methods have been widely used, they are susceptible to numerous external influences, which complicates the precise determination of the time of death. ^3^

Owing to these limitations, forensic practitioners have shifted attention to histological examination and blood-based biochemical assays, which often yield more reliable estimates of the TSD than external findings alone. ^4^

Bone marrow has some unique advantages when it comes to postmortem studies, due to its protected anatomical location and slower rate of decomposition compared to soft tissues. This protected setting helps preserve the blood-forming (hematopoietic) cells inside, making bone marrow a valuable resource for understanding what happened after death. ^5^

After death, the cells in the bone marrow start to break down in a predictable way, offering important clues for estimating the time since death. Once life stops, natural self-digesting (autolytic) processes start, disrupting the delicate balance of cellular homeostasis. The cytoplasm starts to undergo series of progressive morphological alterations, from early vacuolization to eventual dissolution, providing a biologically plausible progressive framework that may be correlated with elapsed time since death correlated with elapsed time since death. ^6^

Understanding the morphological changes of cytoplasmic degeneration in bone marrow cells allows forensic experts to approximate PMI with improved accuracy, especially in the early postmortem period. This study aims to examine these cytoplasmic changes in bone marrow after death and assess their value as reliable biological markers for determining the time since death.

## Materials and methodology

This observational study was designed as a descriptive, cross-sectional investigation conducted from June 2023 to November 2024 in the Department of Forensic Medicine at a tertiary care hospital in Mangalore, India.

Based on a previous study by Biradar G et al. (2016), ^7^ which reported a standard deviation (σ) of 75.66, and using an acceptable margin of error (d) of 13.5 with a 99% confidence interval (Z = 2.58), the minimum calculated sample size was determined to be 43.

The inclusion criteria for this study were cases with a documented time of death based on inquest or hospital records, bodies stored in cold chambers maintained at approximately 4°C before autopsy, no history of hepatological malignancies or bone marrow disorders, and cases where informed consent and to publish clinical details and images was obtained from the legal next of kin of the deceased individuals. The exclusion criteria included cases with a history of hypothermia, those involving burns, neoplasms, or malnutrition, fractures of the sternum, significant pre-mortem blood loss, unknown or disputed time of death, exposure to extreme environmental conditions such as immersion, extreme heat, or freezing temperatures and individuals who had undergone chemotherapy, radiotherapy, or bone marrow transplantation before death.

**Figure 1.**
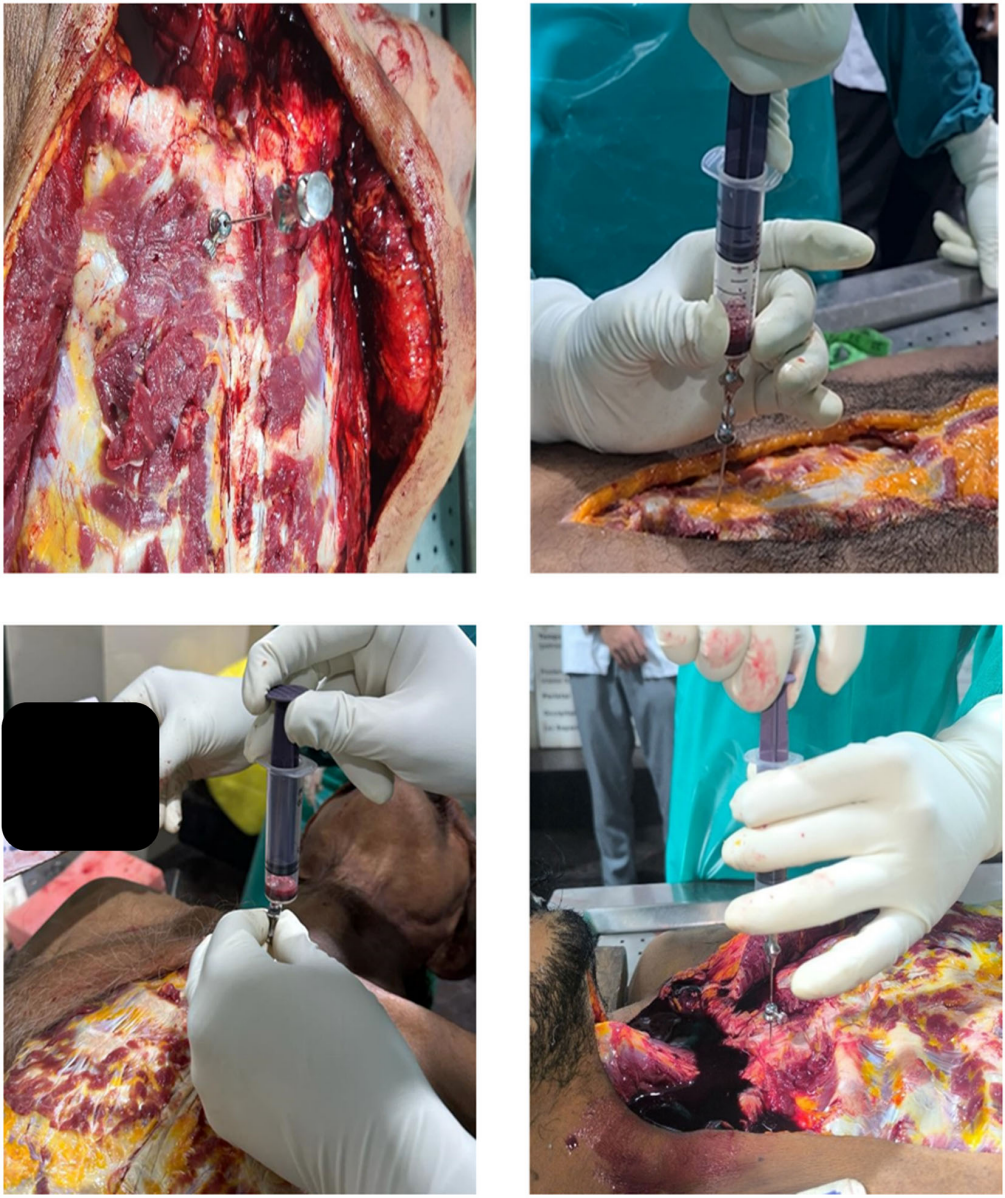
Aspiration procedure - aspirated using a Salah bone marrow aspiration needle.

Sample collection was carried out by first making an I-shaped incision according to standard medico-legal autopsy protocols, carefully exposing the sternum to avoid injury to major vessels. Bone marrow was then aspirated from the junction of the first and second parts of the sternum using a Salah bone marrow aspiration needle attached to either a 10 ml or 50 ml syringe, depending on the resistance ( Figure 1). The aspirated samples were promptly transferred into EDTA tubes to prevent clotting. For smear preparation and staining, the samples were placed on frosted slides using a sterile pipette and spread evenly to create uniform smears. Multiple smears were prepared for thorough analysis. These slides were air-dried at room temperature, fixed with absolute alcohol, and subsequently stained using Leishman stain (Romanowsky technique) to demonstrate cytoplasmic details. During microscopic examination, the stained slides were evaluated under an electronic compound microscope, with particular attention given to cytoplasmic changes such as membrane breaks, vacuolation, and eventual dissolution. All bone marrow smears were examined and scored by a single observer to maintain uniformity; inter- and intra-observer reliability testing was not performed.

**Table 1.** Scoring system for cytoplasmic changes. ^7^

Score	Cytoplasmic changes
0	No observable changes in the cytoplasmic membrane.
1	Breaks or discontinuities in the membrane.
2	Small cytoplasmic vacuoles present.
3	Large vacuoles indicating advanced degeneration.
4	Complete loss of cytoplasmic structure.

The scoring system used to assess cellular changes in the bone marrow was adopted from the study by *Biradar G, SatishBabu BS, Bakkannavar S, Pradeep Kumar G, Shaila B. Estimation of time since death from cytoplasm changes of bone marrow cells (2016)”.* ^7^

### Ethical consideration

This study was conducted after obtaining approval from the Institutional Ethics Committee, K. S. Hegde Medical Academy, Nitte (Deemed to be University), Mangalore (Approval No: INST.EC/EC/131/2023, REG. NO: EC/NEW/INST/2022/KA/0174, dated 15th May 2023).

**Consent to publish:** Written informed consent to publish clinical details and images was obtained from the legal next of kin of the deceased individuals who are included in this study. Consent was granted to the authors by the next of kin, and all efforts were made to anonymize data and images.

### Data collection

The time of death issued by the treating physician was collected from the hospital file.

Data entry and coding were done in Microsoft Excel.

### Data analysis

Descriptive analysis such as frequencies, percentages, mean and standard deviation was done. Statistical analysis was performed using SPSS version 25. The Analysis of Variance (ANOVA) test was conducted to determine whether there were statistically significant differences among the means of multiple groups. p<0.05 considered as statistically significant.

## Results

A total of 43 cases were included in this study, with a clear predominance of males (79.1%) compared to females (20.9%) ( Table 2).

**Table 2.** Gender wise distribution of the participants (N=43).

Gender	N	%
Female	9	20.9
Male	34	79.1
Total	43	100.0

The mean age of the cases was 45.67 years with a standard deviation of 16.21. The mean time difference was 12.74 with a standard deviation of 6.02 ( Table 3).

**Table 3.** Descriptive statistics for age and time difference.

	N	Minimum	Maximum	Mean	Std. Deviation
Age	43	17.0	77.0	45.674	16.2095
Time difference	43	3	26	12.74	6.022

Overall, road traffic accidents (51.2%) were the most common cause of death among the cases, followed by fall from height (20.90%), poisoning (14%) and the least common was sudden death (13.90%).

**Figure 2.**
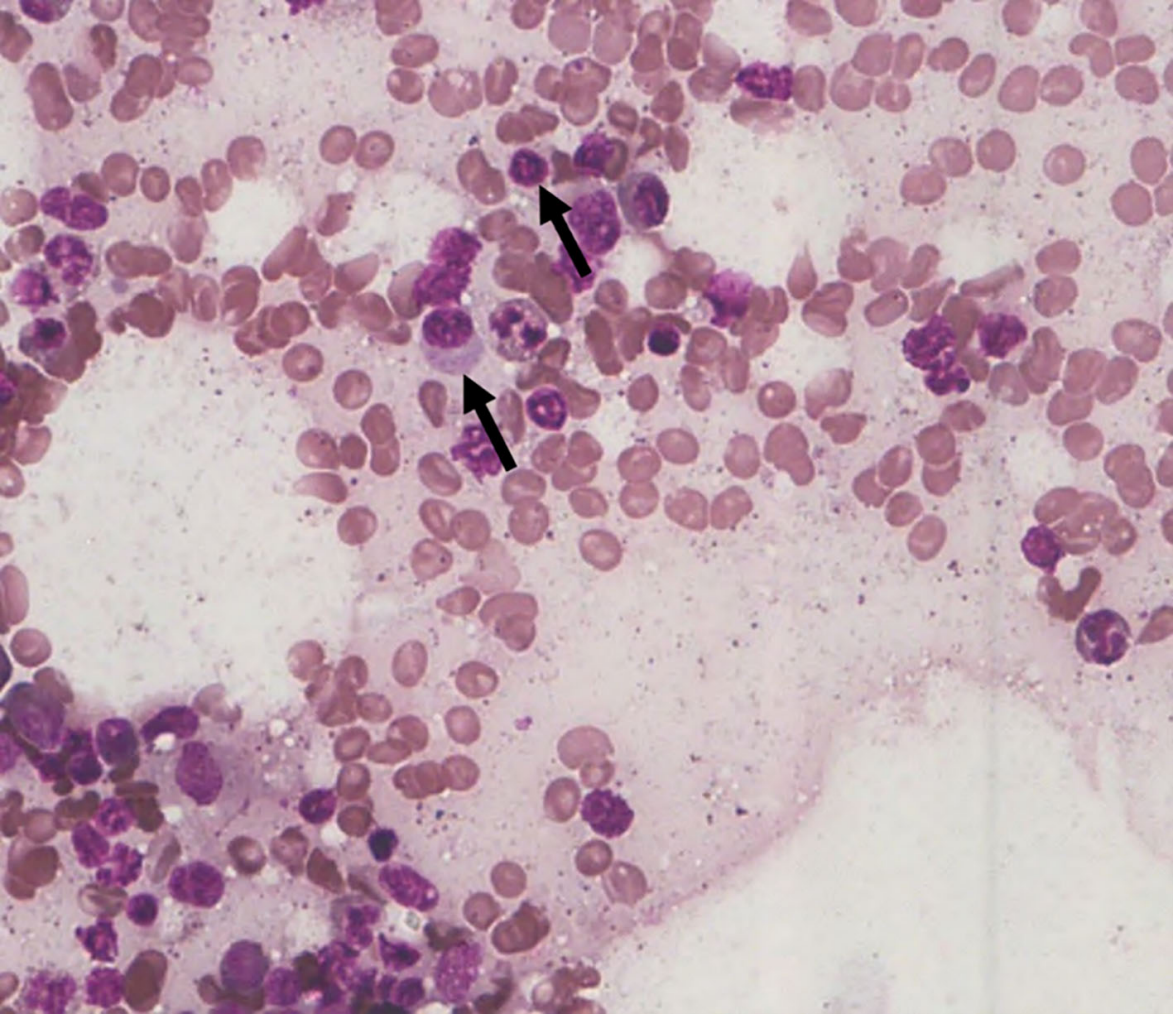
S0 - No change in the cytoplasmic membrane.

**Figure 3.**
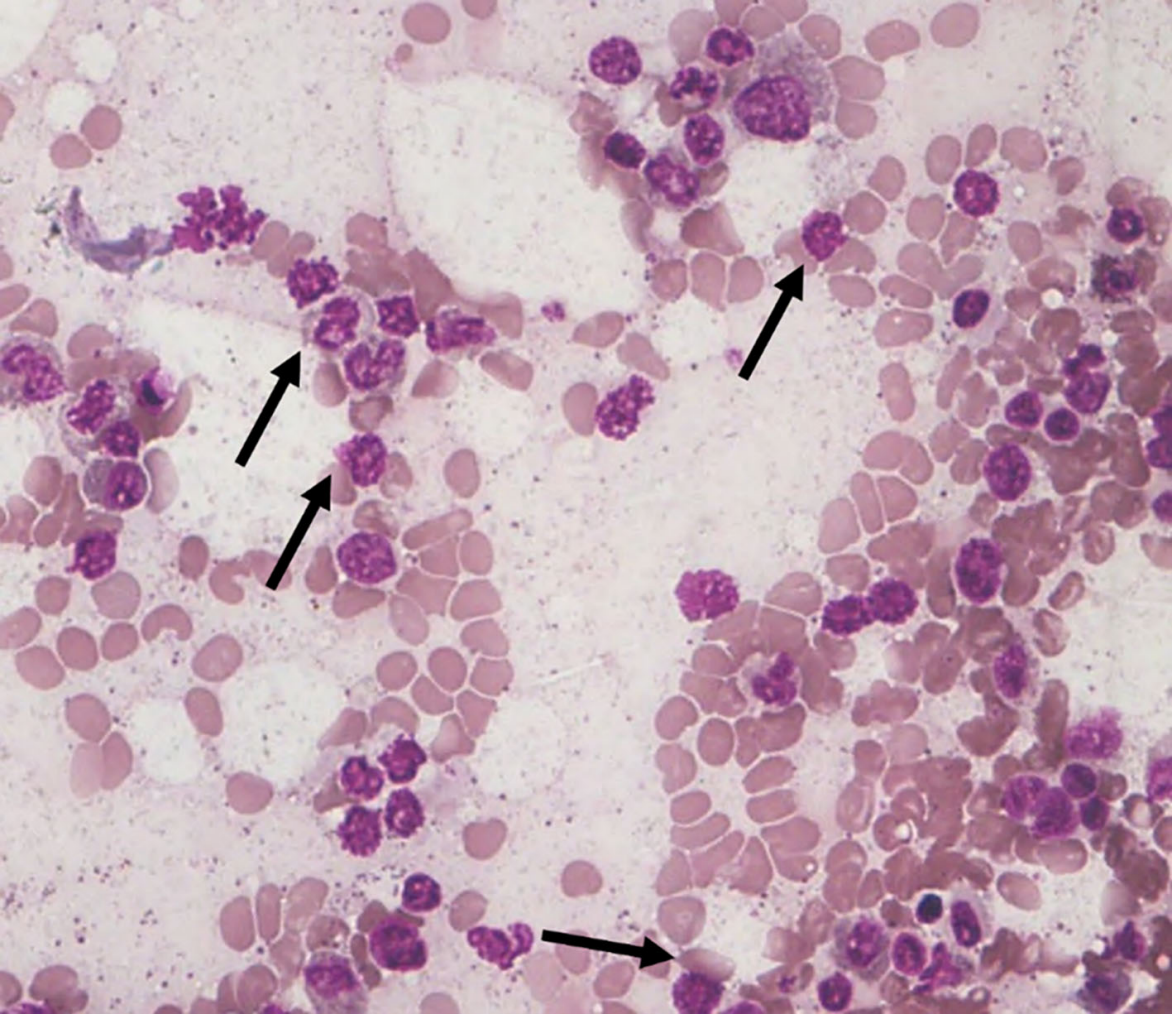
S1 - Break in the cytoplasmic membrane.

**Figure 4.**
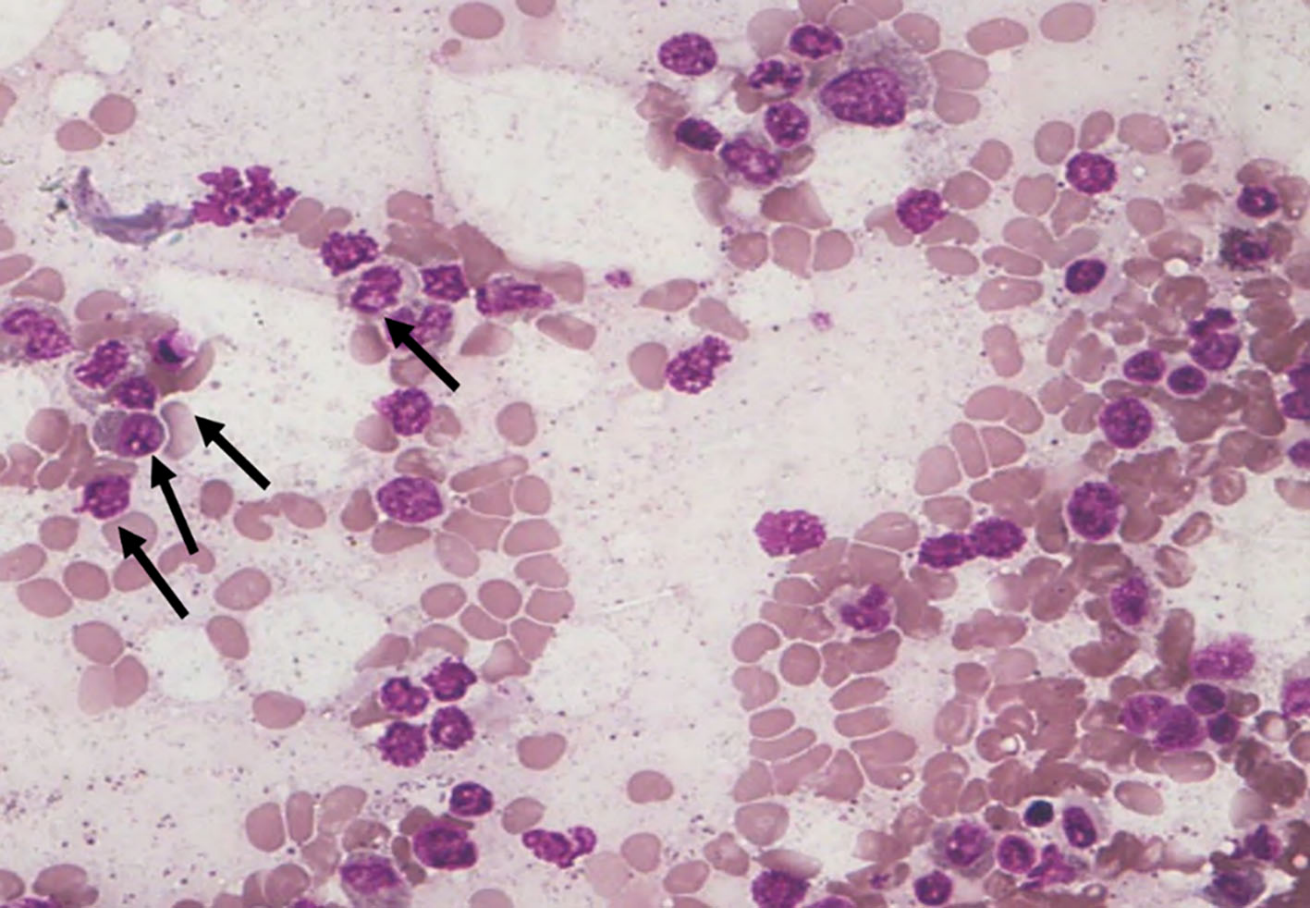
S2 - Small vacuoles in cytoplasm.

**Figure 5.**
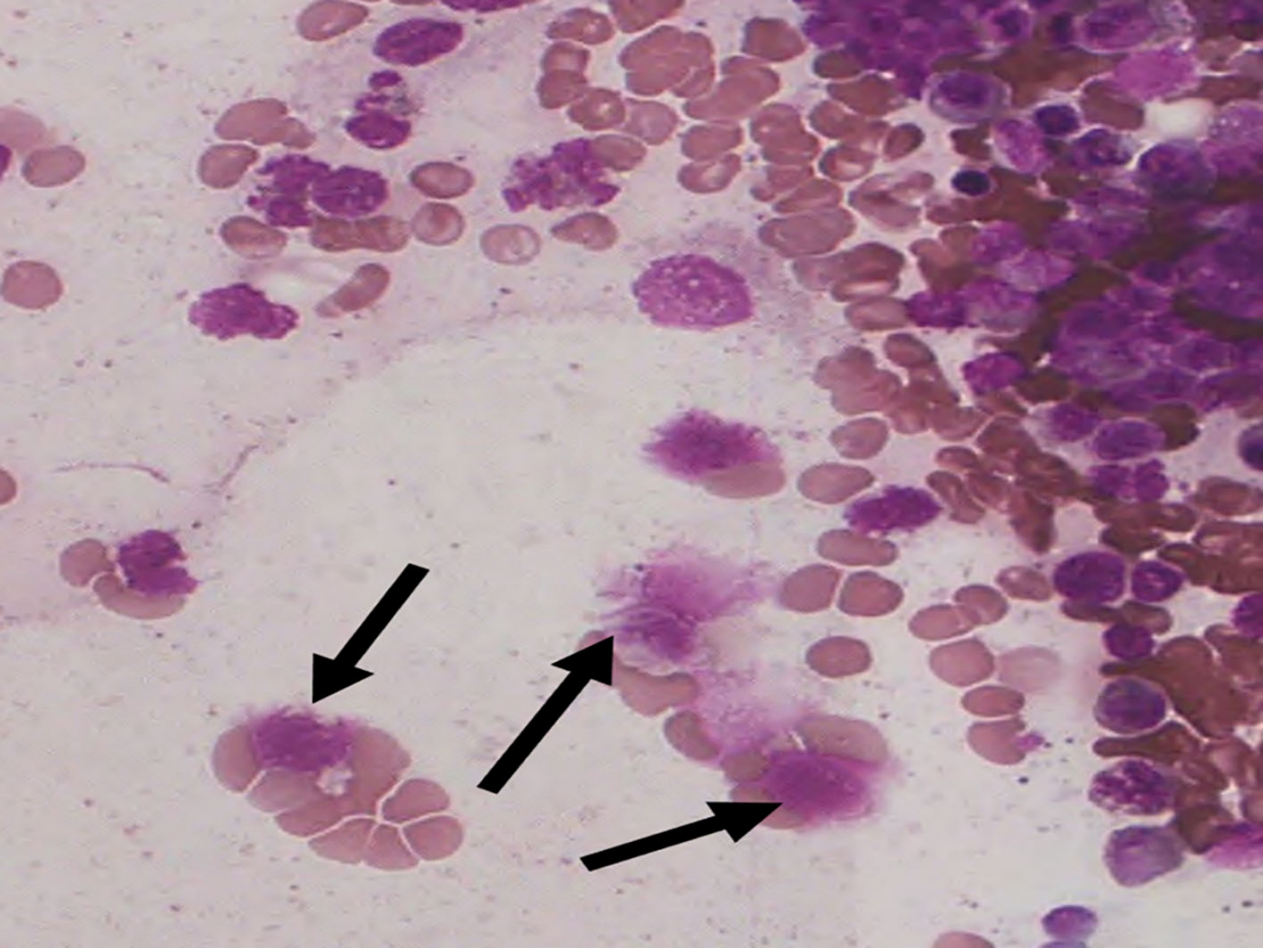
S4 - Complete loss of cytoplasm.

Among the 43 cases studied, cytoplasmic alterations in the bone marrow cells were graded based on the established scoring system. As shown in Table 4. The majority of cases (51.1%) exhibited **mild cytoplasmic changes (S1)** ( Figure 3). A smaller proportion (25.6%) demonstrated **moderate changes (S2)** ( Figure 4), while **extensive cytoplasmic dissolution (S4)** ( Figure 5) was observed in **14.0%** of the cases. Notably, **9.3% of the cases (4 out of 43)** showed **no observable cytoplasmic alterations (S0)** ( Figure 2). No cases demonstrated large vacuolation (S3 stage), hence this category is not represented in the figures.

**Table 4.** Distribution of cytoplasmic changes observed among the cases (N=43).

Cytoplasm changes	Frequency	Percent
S0	4	9.3
S1	22	51.1
S2	11	25.6
S4	6	14.0
Total	43	100.0

An analysis was performed to examine the relationship between the degree of cytoplasmic changes and the time difference (used as an estimate of PMI), as presented in Table 5.

• The four cases classified under S0 (no cytoplasmic changes) had the shortest mean time difference, averaging 8.50 ± 6.56 hours, with a range from 3 to 18 hours.
• The 21 cases with mild cytoplasmic changes (S1) had a mean time difference of 14.09 ± 6.34 hours, ranging from 6 to 26 hours.
• The 11 cases showing moderate changes (S2) displayed a mean of 10.48 ± 4.31 hours, spanning 5 to 17 hours.
• The 6 cases with extensive cytoplasmic changes (S4) had the longest mean time difference, recorded at 14.97 ± 5.84 hours, with values ranging from 7 to 22 hours.

**Table 5.** Relationship between time difference and cytoplasmic changes. (p<0.05 **).

Cytoplasmic changes	N	Mean	Std. Deviation (p)	Minimum	Maximum
S0	4	8.50	6.557	3	18
S1	22	14.09	6.344	6	26
S2	11	10.48	4.307	5	17
S4	6	14.97	5.849	7	22
Total	43	12.74	6.022	3	26

** Statistically significant.

While a trend suggesting increasing cytoplasmic changes with longer post-mortem intervals was noted, variability was observed across categories, and no statistically significant correlation was found between time difference and cytoplasm changes (p>0.05).

## Discussion

The estimation of the post-mortem interval (PMI) remains a cornerstone of forensic practice, guiding both legal and investigative processes. While traditional markers such as rigor mortis, hypostasis, and core body temperature are routinely employed, their reliability diminishes under certain circumstances, including when bodies have been refrigerated or environmental conditions vary significantly. In this context, microscopic evaluation of internal tissues, particularly bone marrow, the primary hematopoietic site, is relatively protected and decomposes more slowly than most soft tissues, so its cytoplasmic and nuclear autolytic changes provide a sequential, biologically plausible framework for PMI estimation. ^8^

Tattoli et al. (2014) emphasised the slower autolysis of bone marrow compared to other tissues, highlighting its potential utility in PMI estimation, especially in the early post-mortem period when traditional markers may yield equivocal results. Although our study did not find a statistically significant correlation, the observable pattern of progressive cytoplasmic changes supports this notion, reinforcing the potential role of bone marrow cytology as a complementary tool in forensic time since death estimation. ^9^

Biradar et al. (2016) observed the onset of cytoplasmic vacuolation at 5–7 hours postmortem, with advanced lysis beyond 16 hours. ^7^ Our study aligns with this timeline, showing mild cytoplasmic changes (S1) in over half the cases (51.1%) and extensive dissolution (S4) most commonly beyond 14–15 hours.

Similarly, Babu et al. (2015) documented progressive vacuolation and cytoplasmic breakdown with increasing PMI, findings echoed in the present work. ^10^ More recently, Sakr et al. (2024) described minimal cytoplasmic changes at 6 hours, with substantial loss of cellular detail by 10–12 hours and necrosis beyond 18 hours. ^11^ The present results concur, showing that while early PMI cases exhibited intact or mildly altered cytoplasm, progressive vacuolation and dissolution became evident after 12 hours.

Michalova et al. (2011) further demonstrated that hematopoietic stem cells remained viable for 2–12 hours within the intact femur, with marrow cell suspensions preserving viability for up to two days at 37°C and four days at 4°C. ^12^ Although our study did not examine stem cell–specific survival, the general cytoplasmic changes we observed beginning at 6–12 hours and advancing by 20 hours are consistent with these findings. Taken together, this highlights the influence of storage conditions, particularly refrigeration, in delaying marrow cell autolysis and thereby extending the preservation window for cytoplasmic morphology.

Although a general trend of increasing cytoplasmic alteration with longer PMIs was observed, statistical correlation was not significant. This variability is likely attributable to confounding factors such as environmental conditions, cold storage, cause of death, and individual biological differences, all of which influence the rate of cytoplasmic autolysis. Nevertheless, the consistency of trends across multiple studies reinforces the potential of cytoplasmic changes as supportive markers for PMI estimation, particularly in the early postmortem period when external indicators are unreliable.

## Conclusion

This study observes a gradual progression of cytoplasmic autolysis in sternal bone marrow cells with increasing post-mortem intervals, from early vacuolation to complete dissolution. Although no statistically significant correlation with PMI was established, likely due to biological variability, environmental influences, and a modest sample size, the observed trends highlight bone marrow cytology as a potential supportive adjunct for PMI estimation. Its simplicity, cost-effectiveness, and relative resistance of bone marrow to decomposition, cytoplasmic evaluation can strengthen forensic assessments when interpreted alongside other morphological, biochemical, and circumstantial evidence to strengthen accuracy and medico-legal reliability.

### Limitations

The study was limited by a small sample size, a single-centre setting, and restriction to sternal marrow, which may limit generalizability. Variability in refrigeration, environmental factors, and individual biological differences also influenced cytoplasmic autolysis. Additionally, interobserver subjectivity in smear interpretation may have introduced variability.

### Recommendations

Future studies should adopt standardised scoring systems, larger and multicentric samples, and comparative analyses with other organs. Incorporating molecular tests, enzyme assays, and immunohistochemistry may improve sensitivity and precision. Such refinements could enhance the reliability of bone marrow cytology as a supportive tool for postmortem interval estimation.

## Data Availability

**BioStudies: Cytoplasmic changes in bone marrow cells as indicators of postmortem interval. S-BSST2159** https://www.ebi.ac.uk/biostudies/studies/S-BSST2159. DOI: 10.6019/S-BSST2159. ^13^ This project contains the following underlying data:
•
**CYTOPLASM DATA SHEET.xlsx** (raw case-wise data including demographic details, cause of death, refrigeration status, Sample collection date and time, postmortem interval, and graded cytoplasmic changes). Data are available under the terms of the Creative Commons Zero “No rights reserved” data waiver (CC0 1.0 Public domain dedication). Cytoplasmic changes in bone marrow cells as indicators of postmortem interval. BioStudies, S-BSST2159. Retrieved from https://www.ebi.ac.uk/biostudies/studies/S-BSST2159. ^13^

## Software availability

No specialized software was required beyond Microsoft Excel (2021) for dataset compilation. Data analysis was conducted using SPSS (version 25).
