# Supplementary material for: Cytoplasmic changes in bone marrow cells as indicators of postmortem interval
Source: F1000Res. 2025 Dec 26;14:964. Originally published 2025 Sep 22. [Version 2] doi: 10.12688/f1000research.169755.2 (PMC12856251; doi:10.12688/f1000research.169755.2)
Supplement: Peer review history

# Reviewer report: Reviewer response for version 2

**DOI:** 10.5256/f1000research.194256.r445335

**Published:** 2026-01-29

**Authors:** Matteo Nioi

**Affiliations:** 1 University of Cagliari, Cagliari, Italy

This manuscript presents a descriptive, cross-sectional forensic pathology study investigating cytoplasmic autolytic changes in sternal bone marrow cells as a potential correlate of the postmortem interval (PMI). The authors analyzed 43 medico-legal autopsy cases with documented times of death, applying a predefined cytoplasmic scoring system (0–4) on Leishman-stained bone marrow aspirates.

 The study addresses an important and underexplored topic in PMI estimation, namely the use of bone marrow cytology, a tissue that is anatomically protected and potentially less susceptible to early postmortem degradation compared to external tissues. The methodology is clearly described, ethical aspects are appropriately handled, and all underlying data are openly available. The revised version adopts a more cautious and appropriate interpretative tone, explicitly acknowledging the lack of statistical significance and the exploratory nature of the findings.

 Overall, the work represents a hypothesis-generating investigation rather than a validated forensic method. To further improve scientific clarity, robustness, and alignment between data and interpretation, the authors are encouraged to address the following points:

**1)** explicitly classify the study throughout the manuscript (title, abstract, discussion, and conclusions) as a *proof-of-concept / exploratory study*, clearly stating that it is intended for hypothesis generation and not for validation or prediction of PMI;

**2)** ensure that the absence of statistical significance (p > 0.05) is consistently emphasised in the abstract and conclusions, avoiding any wording that may imply sequential reliability or standalone forensic applicability;

**3)** expand and clarify the discussion of refrigeration as a major confounding variable, explicitly stating that individual cold-storage duration was not stratified or analysed and explaining how this limitation may have influenced autolytic progression and score distribution;

**4)** interpret the observed non-linear pattern, including the inversion between intermediate cytoplasmic scores, as a methodological and environmental artefact rather than as a biological phenomenon;

**5)** acknowledge more explicitly the limitations related to single-observer scoring and the absence of inter- and intra-observer reliability assessment, and recommend blinded multi-observer evaluation in future studies to improve reproducibility;

**6)** frame the statistical analysis strictly as descriptive, clarifying that the study was not powered for inferential modelling, and suggest that future investigations adopt a priori power calculations and regression-based or mixed-effects approaches incorporating relevant confounders;

**7)** reinforce in the final conclusions that the principal contribution of the study lies in demonstrating feasibility and biological plausibility of bone marrow cytoplasmic assessment, rather than in proposing a validated forensic tool, and outline concrete directions for future multicentric and methodologically controlled research.

Is the work clearly and accurately presented and does it cite the current literature?

Yes

If applicable, is the statistical analysis and its interpretation appropriate?

Partly

Are all the source data underlying the results available to ensure full reproducibility?

Yes

Is the study design appropriate and is the work technically sound?

Yes

Are the conclusions drawn adequately supported by the results?

Partly

Are sufficient details of methods and analysis provided to allow replication by others?

Partly

Reviewer Expertise:

Forensic pathology and legal medicine, with expertise in postmortem interval assessment, postmortem imaging, and determination of cause of death.

I confirm that I have read this submission and believe that I have an appropriate level of expertise to confirm that it is of an acceptable scientific standard, however I have significant reservations, as outlined above.

# Reviewer report: Reviewer response for version 1

**DOI:** 10.5256/f1000research.187128.r425571

**Published:** 2025-11-12

**Authors:** Shankar Bakkannavar

**Affiliations:** 1 Kasturba Medical College Hospital, Manipal Academy of Higher Education, Manipal, India

**Overall comments:**

 The manuscript is well written addresses an important and less explored area of forensic pathology wherein bone marrow cytology is used for postmortem interval (PMI) estimation. It demonstrates clear structure, methodological transparency, and responsible scientific reporting. Improvements in methodological detail, data interpretation, and contextual framing are necessary to enhance its scientific impact and clarity.

- The title is concise, informative, and well-aligned with the content.
- The abstract presents the aim, methods, and key findings clearly. However, the conclusion in the abstract should better reflect the *non-significant* statistical findings. Consider including confidence intervals or p-values in the abstract for transparency.
- The introduction provides a logical flow from traditional PMI estimation techniques to the rationale for bone marrow analysis. Integrate recent molecular and biochemical PMI estimation studies (e.g., protein degradation, mRNA decay, oxidative biomarkers). Add a brief paragraph clarifying *why cytoplasmic changes*, as opposed to nuclear or biochemical ones, may have practical forensic utility.

- Methodology is detailed, especially regarding sampling and staining procedures. Ethical considerations and inclusion/exclusion criteria are well described.  
- However, specify storage temperature and duration for each case, as this significantly affects autolysis.
- State whether scoring was performed by a single or multiple blinded observers
- Though ANOVA is used here, regression or correlation analysis might be more appropriate for continuous PMI data.

- Tables and figures are clear and appropriately labelled. 

- Discussion is well written which compares findings with multiple previous studies 
- Biological reasoning (autolytic sequence, environmental effects) is well explained.
- Expand on the possible reasons for statistical non-significance (small sample, wide variance, temperature confounding).

- Incorporate the recent reviews and systematic studies

Is the work clearly and accurately presented and does it cite the current literature?

Yes

If applicable, is the statistical analysis and its interpretation appropriate?

Yes

Are all the source data underlying the results available to ensure full reproducibility?

Yes

Is the study design appropriate and is the work technically sound?

Yes

Are the conclusions drawn adequately supported by the results?

Yes

Are sufficient details of methods and analysis provided to allow replication by others?

Yes

Reviewer Expertise:

Forensic Pathology, Identification of human beings, Toxicology

I confirm that I have read this submission and believe that I have an appropriate level of expertise to confirm that it is of an acceptable scientific standard.

# Reviewer report: Reviewer response for version 1

**DOI:** 10.5256/f1000research.187128.r420291

**Published:** 2025-10-28

**Authors:** Simona Sulis, Lenka Vorobeľová

**Affiliations:** 1 Department of Anthropology, Comenius University Bratislava, Bratislava, Slovakia

This descriptive cross-sectional study investigates the relationship between cytoplasmic autolytic changes in sternal bone marrow cells and postmortem interval estimation. The authors analysed 43 medico-legal autopsy cases with recorded times of death, scoring cytoplasmic changes from 0 (intact morphology) to 4 (complete dissolution) using Leishman-stained bone marrow aspirates. Results showed a predominance of mild cytoplasmic alterations (51.1%), with mean PMI values ranging from 8.5 hours (S0) to 15.0 hours (S4). However, the correlation between cytoplasmic score and PMI did not reach statistical significance, and the progression showed inconsistencies, notably with Score 2 displaying a lower mean PMI than Score 1.

 The manuscript displays good structure and clarity. The introduction provides a solid rationale by addressing the limitations of traditional PMI markers and highlighting bone marrow as a promising alternative substrate because of its protected anatomical position. The literature review suitably references recent studies. However, the discussion could be enhanced by broader contextualisation within current forensic methods, especially molecular and biochemical markers for PMI estimation. Including more references that compare histological techniques with larger sample sizes and advanced statistical methods would better position the manuscript within the existing research landscape.

 Some suggested references:

 [(Sakr, et al., 2024) - ref 1]

 [(Franceschetti, et al., 2023) - ref 2]

 [(Weisensee, et al., 2024) - ref 3]

 [(Zissler, et al., 2020) - ref 4]

 The inclusion and exclusion criteria are clearly defined, appropriately excluding confounding factors such as burns, malnutrition, chemotherapy, and extreme environmental exposure. However, several limitations in the design need attention. The single-centre approach limits generalisability, and the manuscript would benefit from a discussion of how regional factors (climate, storage protocols) might influence the results.

 While the methodology section provides adequate detail on specimen collection, staining procedures, and the scoring system, some gaps undermine reproducibility. Most notably, the manuscript acknowledges refrigeration of bodies prior to autopsy but fails to systematically record the temperature and duration of cold storage for individual cases. This is a confounding variable, as refrigeration significantly slows autolytic processes. The unusual observation that Scores 2 has a lower mean post-mortem interval than Score 1 may directly stem from varying refrigeration conditions. Additional methodological details needing clarification include, for example, whether scoring was performed by a single observer or multiple blinded reviewers, and whether inter-observer or intra-observer reliability testing was carried out.

 In the discussion, the authors should address the non-linear pattern and its implications more thoroughly, expand the discussion of refrigeration's confounding effects with potential stratified analysis, compare findings more critically with cited studies, and broaden the contextualisation within contemporary forensic approaches, including molecular and biochemical markers.

 The conclusions need significant adjustment to match the statistical results. The authors describe cytoplasmic scoring as a "useful adjunct" and state that "cytoplasmic autolysis in bone marrow follows a sequential pattern," but the data do not support these claims. The lack of statistical significance, along with the unusual S1/S2 inversion, questions the validity of claims about a clear sequential progression. Without proven predictive value or validation in an independent sample, statements about practical usefulness are premature.

 The authors rightly acknowledge the non-significant results and overlap between categories, and the limitations section honestly discusses sample size, single-centre design, and environmental confounders. However, the conclusions should better reflect the exploratory nature of this study. A more cautious statement would note that although trends warrant further investigation, the current evidence does not yet statistically confirm the method's reliability or standalone application. The considerable biological variability and methodological limitations that hindered the detection of significant associations should be given greater emphasis.

 Overall, this study tackles an important question in forensic pathology by examining a relatively unexplored tissue with potential benefits for PMI estimation. The research question is valid, the methodology is mainly sound, and the authors show scientific integrity through transparent reporting and open data sharing. However, the manuscript needs revision to address statistical issues, methodological gaps (especially concerning refrigeration documentation and controls), and conclusions that potentially exaggerate the findings. With specific revisions to address these points, this work could offer a strong basis for future research and contribute significantly to the forensic literature on postmortem interval estimation.

Is the work clearly and accurately presented and does it cite the current literature?

Yes

If applicable, is the statistical analysis and its interpretation appropriate?

Partly

Are all the source data underlying the results available to ensure full reproducibility?

Yes

Is the study design appropriate and is the work technically sound?

Yes

Are the conclusions drawn adequately supported by the results?

Partly

Are sufficient details of methods and analysis provided to allow replication by others?

Partly

Reviewer Expertise:

Forensic anthropology, biomedical anthropology

We confirm that we have read this submission and believe that we have an appropriate level of expertise to confirm that it is of an acceptable scientific standard, however we have significant reservations, as outlined above.

## References

[1] : Estimation of postmortem interval using histological and oxidative biomarkers in human bone marrow. *Forensic Science, Medicine and Pathology* .2023;20(3) : 10.1007/s12024-023-00753-9 910-919 10.1007/s12024-023-00753-9 37987965

[2] : Estimation of Late Postmortem Interval: Where Do We Stand? A Literature Review. *Biology* .2023;12(6) : 10.3390/biology12060783 10.3390/biology12060783 PMC1029526637372068

[3] : Human Decomposition and Time Since Death: Persistent Challenges and Future Directions of Postmortem Interval Estimation in Forensic Anthropology. *American Journal of Biological Anthropology* .2024;186(S78) : 10.1002/ajpa.70011 10.1002/ajpa.70011 PMC1189855540071863

[4] : Postmortem Protein Degradation as a Tool to Estimate the PMI: A Systematic Review. *Diagnostics* .2020;10(12) : 10.3390/diagnostics10121014 10.3390/diagnostics10121014 PMC776077533256203

# Response

**Published:** 2025-11-11

**Authors:** Vinoth Guru

**Author Response**

 We thank the reviewers for their constructive feedback and valuable observations.

 This study was specifically designed to examine histopathological (cytoplasmic) changes in bone marrow cells in relation to postmortem interval, as stated in the title and objectives. Therefore, molecular and biochemical methods were beyond the intended scope of this work.

 All bone marrow smears were examined and scored by a single observer to maintain uniformity and reduce inter-observer bias. Inter- and intra-observer variation testing was not performed. All bodies were kept in the mortuary cold chamber maintained at approximately 4°C, which is standard in Indian mortuary facilities.

 The dataset includes documented times of death, placement in cold storage, and sample collection for each case; however, the specific duration of refrigeration was not analysed separately, which has been acknowledged as a limitation.

 The possible confounding effect of refrigeration on autolysis has been recognised in the Limitations section. The minor inversion between Scores 1 and 2 likely reflects refrigeration variability, sample size constraints, and biological variation, and is interpreted as a methodological variation rather than a biological reversal.

 The ANOVA results showed no statistically significant correlation (p > 0.05) between cytoplasmic score and postmortem interval. The significance threshold (p < 0.05) was indicated in Table 5, and results were reported as non-significant. As the analysis was primarily descriptive, exact F-values and degrees of freedom were not included but can be provided if required.

 The findings are preliminary and exploratory, not confirmatory. The terms “appears,” “suggests,” and “may serve” were intentionally used to indicate trends rather than established relationships. The study emphasises the potential value of bone marrow cytoplasmic changes as a supportive parameter for postmortem interval estimation while acknowledging biological, environmental, and methodological limitations.

 In conclusion, the current version of the manuscript presents an exploratory interpretation, offering preliminary evidence that warrants further validation through larger, multicentric, and molecular correlation studies.
